# Is Biofeedback through an Intra-Aural Device an Effective Method to Treat Bruxism? Case Series and Initial Experience

**DOI:** 10.3390/ijerph18010051

**Published:** 2020-12-23

**Authors:** Kira Pfeiffer, Thaqif El Khassawna, Deeksha Malhan, Christine Langer, Barbara Sommer, Mohamed Mekhemar, Hans-Peter Howaldt, Sameh Attia

**Affiliations:** 1 Department of Cranio Maxillofacial Surgery, Justus-Liebig University Giessen, Klinik Str. 33, 35392 Giessen, Germany; Kira.Pfeiffer@dentist.med.uni-giessen.de (K.P.); HP.Howaldt@uniklinikum-giessen.de (H.-P.H.); 2 Experimental Trauma Surgery, Faculty of Medicine, Justus-Liebig University of Giessen, Aulweg 128, 35392 Giessen, Germany; thaqif.elkhassawna@chiru.med.uni-giessen.de; 3 Institute for Theoretical Biology, Charite Unversitätmedizin Berlin, Invaliedenstr. 110, 10115 Berlin, Germany; deeksha.malhan@charite.de; 4 Department of Otorhinolaryngology, Justus-Liebig University Giessen, Klinik Str. 33, 35392 Giessen, Germany; christine.langer@hno.med.uni-giessen.de (C.L.); Barbara.Sommer@hno.med.uni-giessen.de (B.S.); 5 Clinic for Conservative Dentistry and Periodontology, School of Dental Medicine, Christian Albrechts-Universität zu Kiel, Arnold-Heller-Str. 3, Haus 26, 24105 Kiel, Germany; mekhemar@konspar.uni-kiel.de

**Keywords:** biofeedback, TMD, bruxism, Intra-aural devices

## Abstract

Biofeedback was reported as an effective concept for bruxism treatment, through increasing patient’s awareness of the habit. During bruxing both ear canals become tighter, therefore, an in-ear device can provide biofeedback. The in-ear device is fitted to the ear canal in physiological status, during bruxing the ear-canal tightens resulting in stress on the canal walls and unpleasant feeling. Subsequently, patients stop their bruxing habit. The aim of this study is to provide first clinical evidence that in-ear devices have a positive impact on relieving bruxism in patients. Despite the low number of patients, this early study was designed as a controlled prospective study. The trial included seven female patients with a median age of 47.3 years (23–64 years). Only two patients implemented their devices for eight and seven months, respectively. One patient reported a relief in her symptoms, like headaches and pain intensity during the night, by 50% after three month and 80% after six months. Despite the limited number of participants, the study reflects a potential of Intra-aural devices as effective biofeedback devices in treating bruxism.

## 1. Introduction

Bruxism is defined as a repetitive jaw-muscle activity caused by grinding and/or clenching of the teeth. The disease has two circadian manifestations in either wakeful periods (wake bruxism) and/or during sleep, which is indicated as sleep bruxism [1,2,3]. Temporomandibular joint disorder (TMD) is a collective term for degenerative musculoskeletal conditions associated with functional and morphological impairments resulting mainly in patients suffered from bruxism [4]. TMD includes deformities of the disc position and/or structure within the temporomandibular joint and its associated musculature [5]. Clinical symptoms of TMD varied between painful sounds at the joints region, restricted or deviating of the jaw movement, orofacial pain and muscular pain [6]. Literature presented that 16–59% of the population show symptoms of a TMD [7]. However, neither etiology nor progression are quite understood. However, surgical and non-surgical treatment choices are directed to reducing pain, rather than addressing the etiology [8,9]. The intent of a treatment method is to stabilize the temporo-mandibular joint, protect the teeth, to relax the muscles and decrease pressing or clenching. Nevertheless, Scrivani et al. claim 85–90% of temporomandibular disorders (TMD) can be treated with non-surgical, non-invasive and reversible therapeutic methods [10]. Although with no clear scientific explanation, TMD is associated to bruxism, which is consequential on natural dentation and dental implants [11]. Therefore, an interest of bruxism from definition and etiology to characterization of biomechanical activities (grinding and clenching) is increasing beside the pain management aspect. Unfortunately, much remains unclear, and interpretation of data based on patient self-report of bruxism should be cautiously taken [12]. In an umbrella review of 41 systemic reviews, prevalence of a wake bruxism in adults population of 22–30%, while the sleep bruxism ranged between 1–15% [13]. Therapy approaches for bruxism include occlusal appliances; pharmacological therapies; biofeedback therapies; and miscellaneous therapies (e.g., prosthetic rehabilitation adenoidectomy) [13]. Additionally, dental or medical treatment choices, behavioral adjustment is a promising treatment approach [14]. An appliance should also create awareness of the habits in patients and change the abnormal clenching of the teeth to a relaxed jaw position. Some types of splints can only be worn during nighttime, especially when devices do not cover the full dental to avoid extrusion of the non-covered teeth [15]. However, nighttime worn splints will not provide help for people suffering from wake bruxism. Occasionally, it has been shown that occlusal splints had a negative impact on the condyle-disk relation of patients who showed a disk displacement with reduction [16]. Therefore, a new low-risk and invisible method to treat both forms of bruxism (wake- and sleep-bruxism) is demanded [15,17]. Essential part of the treatment of bruxism is to inform the patients about their bruxism habit. The increased self-awareness leads to self-observed behavior and enables the patients to be aware of frequency and conditions that cause their bruxism during the day [18,19]. Biofeedback is one method to assert behavior control.

Biofeedback is a self-regulation technique were patients learn voluntary control which was once thought to be involuntary body processes. For that intervention it is required to have specialized equipment to convert physiological signals into visual and auditory cues. A trained biofeedback practitioner should guide the therapy. Amongst medical conditions like epilepsy, anxiety, and chronic pain, biofeedback has also shown efficacious results on people with TMD [20]. Scientific approaches concerning biofeedback in bruxism therapy lead back to 2014 where Gu et al. tested a wireless maxillary splint that created a vibrating signal as soon as a bruxing event occurred. The results showed that biofeedback might be a convenient and effective method for mild bruxers compared to a splint therapy [21]. Another approach on biofeedback in treating bruxism is the Grindcare^®^ device. Patients wear it during the night and it has a built-in biofeedback algorithm, which controls functional electric stimulation (FES), which means it induces controlled low voltage electrical impulses (1–7 mA). These impulses lead to interruption of the muscular activity and, therefore, create local muscle relaxation. It subdues unwanted and potentially harmful muscular activity without waking up the user. In the short-term it should reduce the number of clenching/grinding episodes. In the long-term it is considered that the use of the device accustoms users to the impulse which leads to a reduction of their muscular parafunction [22]. Seeing that both of these devices can only be worn during night-time, it led to the idea of a more invisible method. Invisible in the ear canal devices (IIC) are used as hearing aids for a long time and have proven to be comfortable and invisible [23].

**Figure 1:**
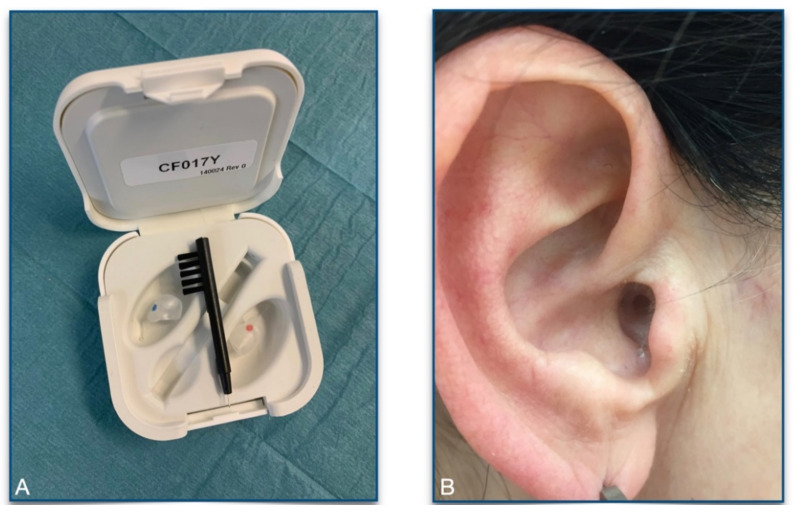
A personalized, compact, and less visible device encourages patients to implement during both circadian cycles of the day. (**A**) Compact Cerezen™ in-ear device kit includes two transparent parts color-coded in correspondence to each ear as well as a cleaning utensil. (**B**) Transparent, personalized ear pods insure low visibility and easy fitting, thus motivating the patient to use them.

Intra-aural devices that work on a biofeedback-basis could be a comfortable treatment concept for bruxism which increase the patient’s awareness of the habit. They are invisible, non-invasive and reversible device to help the patient during wake and sleep phases [24]. Regarding in-ear devices to treat bruxism we found ten patents in the last 40 years which registered for this purpose. However, there was only one product that was tested in a clinical trial and was available for patients to purchase. The name of the product was TMD’es™ (Ascentia Health, Inc., Rockford, IL, USA) which has later been changed to Cerezen™ (Renew Health Limited, Athlone, Ireland) [24]. The device is hollow hard-plastic inserts that fit into the patient’s ear-canal (Figure 1). They can be easily inserted and taken out by the patient and can be worn for up to 23 h a day. When the jaw is in a closed position (bruxing habit), the devices apply pressure on the walls of the ear-canal which induces a biofeedback reaction in users to keep their mandible out of occlusion. This mechanism should minimize clenching or grinding which is supposed to set down the muscle-activity. The initial aim of the pilot study was to examine the effectiveness of this therapeutic method prospectively. Unfortunately, after including seven patients in the study the production of Cerezen™ was stopped in November 2019. Based on the data collected so far, we try to give a statement:

1. If the patients by using such devices be aware of their habit and therefore reduce or stop to bruxism
2. Whether in-ear devices have a positive impact on patients suffering from bruxism or not.

## 2. Materials and Methods

### 2.1. Study Design

The pilot study was designed as a controlled, prospective study at the Department of Oral and Maxillofacial Surgery of the University of Giessen, Germany. The study was approved by the Ethics Committee of the Faculty of Medicine in Justus-Liebig University Giessen, Germany (approval No. 98/19). This study included seven female patients. All patients have been informed about the study and signed the informed consent. Written informed consent for publication of their details was obtained from the study participant. This research has been conducted in accordance with the Declaration of Helsinki. Patients inclusion criteria were: patients with bruxing habits and suffering from cranio-mandibular dysfunction (CMD) according to checklist for temporomandibular joint disorder TMD [25], that were over 18 years old. Patients with one or more of the following (symptoms) were excluded: atypical ear canal shape, hearing aids, physically or mentally unable to use the devices, malignant tumor in head and neck region, during or after radiotherapy, insufficient prosthetic restoration, currently under orthodontic treatment.

### 2.2. Treatment Procedures

**Figure 2:**
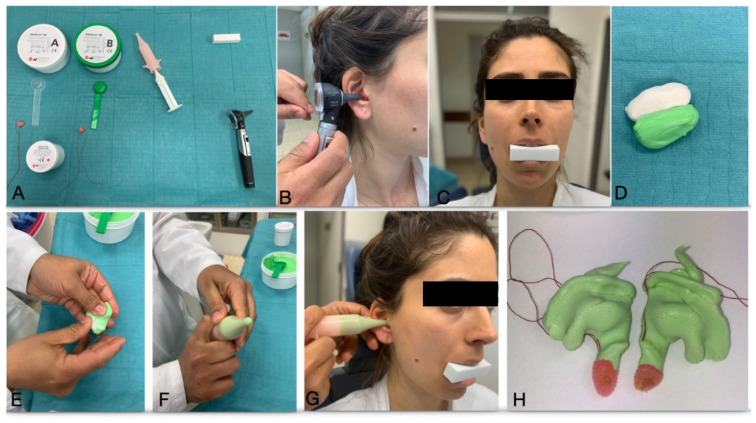
Impression procedure to produce personalized Cerezen in-ear device: (**A**) All materials and instruments needed for taking an impression including: 1—Impression material (Otoform Ak, Dreve Ostoplastik GmbH, Unna, Germany); 2—Impression pads which are a foam cone with thread (Dreve Ostoplastik GmbH, Unna, Germany), 3—Universal syringe to applying the impression material in the ear canal (PC Werth, London, UK); 4—Bite block (Renew Health Limited, Westmeath, Ireland); 5—Otoscope for investigation of the ear canal and insertion of the impression pads in the right position (Heine Optotechnik, Herrsching, Germany) (**B**) Insertion of the impression pads (orang) to protect the ear drum during impression-taking, (**C**) Patient biting on the Bite block to keep the jaw in rest position during the impression-taking (**D**–**F**) Two-components impression materials, Mixing,preparation for injection, (**G**) Impression-taking while patient biting on the Bite Block to fit the impression in the relaxed status (**H**) Ear canal impressions for both sides.

Patients were informed about the device and the treatment procedure as per the rules and guidelines of the University Hospital of Giessen and Marburg. Patients received counsel on the use of the device and possible Ear-Nose-Throat (ENT) specialist examined the patients to exclude any pathological problem in the ear canal. Excessive ear wax was removed and the impression was taken by using Otoform^®^ impression material (Dreve Otoplastik GmbH, Unna, Germany) (Figure 2). The impressions were sent to the company, the devices production took about two weeks. After proper patients’ instruction the in-ear devices were inserted with the following wearing protocol:

- 1st to 3rd: day 3 h/day (only during the day)
- 4th to 7th day: 2 wearing times of 4 h/day (only during the day)
- 2nd week: if no complaints occurred in the 1st week, start from the 2nd week all day (only during the day)
- 3rd week: in consultation with the patient start of wearing also at night

Cerezen(R) can be worn up to 23 h a day. Patients had a two-month testing phase during which they could give back the devices if they didn’t feel improvement of symptoms or if they were dissatisfied with the devices. In this instant patient were reimbursed for the purchase of the device. Patients also had the chance to ask for a refinement of the devices if they felt like the devices did not fit well.

### 2.3. Study Variables

Data about: patients cranio-mandibular dysfunction (CMD), pain behavior and quality of life were recorded before and 3, 6, and 12 months after the treatment by using the following questionnaires: Oral Health Impact Profile (OHIP-21) [26], Oral Behavior Checklist (OBC) [27], as well as a Temporomandibular Disorder Screening Checklist (TMD-Screening) [25] and self-designed examination sheet that also includes the Visual Analogue Scale Score (VAS-Score). The study started in January 2019 till the company stopped the production in November 2019.

### 2.4. Statistical Analysis

Descriptive statistics were performed using Statistical package PASW (version 25.0; IBM Corporation, Armonk, NY, USA). Frequency analysis was carried out and data were shown in bar graphs. Due to the nature of the data and population size no significance testing was possible to perform. The Kaplan–Meier curve was used to exhibit the duration in which patients wore the device.

## 3. Results

Between January and November 2019, we could include seven female patients (ages 23 to 64, median: 47.3) in the study. Two patients used the device for a total of seven and eight months. The fact that all patients were female is just a coincident that is not integral to the study design.

One patient showed a relief in her symptoms like headaches as well as the pain intensity during the night by 50% after three months and 80% after six months. However, side effects as slightly bruised ear-canals were also shown. In this case the patients were recommended to use a lubricant and the side effect was solved. Another side effect was the looseness of the device, in this case the device was reclaimed and the company produced a new one which is better fitting. The other patient reported a lower intensity of her headaches and has the feeling of clenching her teeth less. However, she also noticed muscle tension without pressing increased and by wearing her Cerezen™-devices she indicated hearing a rustling noise while chewing so she did not wear it while she ate. Three patients gave back their devices within the eight-week testing period with no interest of refinement of the devices for a better fit. One patient found that even after the refinement the fit of the devices was uncomfortable and, therefore, gave them back. The other patient felt a rebound after an initial improvement of her symptoms and gave back the devices after the refinement. Table 1 summarizes data of the patients included in this trial.

**Figure 3:**
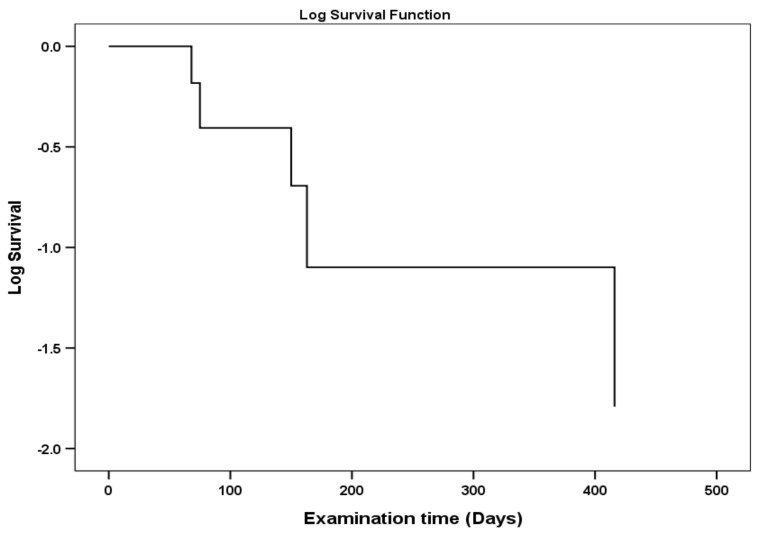
Kaplan–Meier curve asserted the assumption the independence of the data failure. The longest time a patient used the in-ear device was about 400 days.

To describe the time-period that patients used their in-ear devices a Kaplan–Meier estimator was used. The Kaplan–Meier curve showed that the failure in the observed times is independent and therefore, the data consistent and asymptotically normal (Figure 3).

### Improvement of Teeth-Grinding

**Figure 4:**
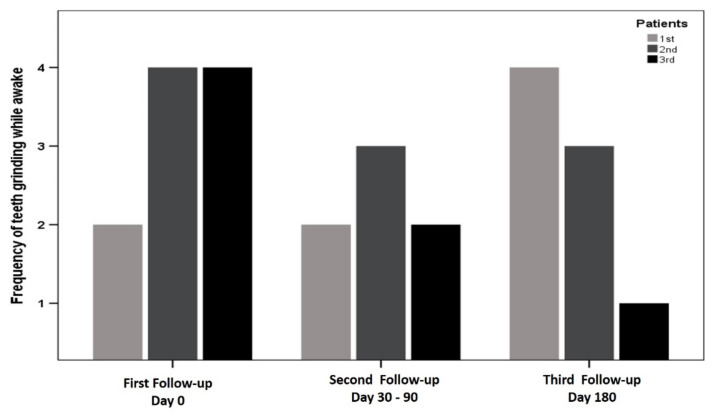
Graphical depiction of changes in frequency of teeth grinding among three patients. Patients two and three showed less grinding with follow-up progression. The results infer the benefit of biofeedback during the wakeful cycle.

Teeth-grinding improved while awake in patient two and three who continued to wear the devices. Patient one gave back the device after five months. Initially she felt significant improvement of her symptoms but observed some relapse after four months which could be seen in her second and third follow-up (Figure 4).

## 4. Discussion

Biofeedback is a well-proved treatment method in the rehabilitation therapy [28]. Biofeedback alerts patients of their bruxing habits in real time [29]. Therefore, feedback models gained more importance over occlusion to treat bruxism [30].

Early in-ear devices to treat bruxism using biofeedback, were bulky and could be worn during the night therefore directed more towards sleep bruxism. Keeping the non-invasive advantage of the device a discrete one can be used during the day and increase the time of feedback. Therefore, device used here is modified in size and is transparent and can be worn for 23 h a day. Despite its advantages such devices were not subjected to controlled clinical studies. At the study beginning patients showed good compatibility and acceptance for the device and were eager to use it. However, minor complications occurred, such as redness and sensations in the ear canal and tinnitus. Patients who decided to give back their devices early on, described unpleasantness during sleeping. However, this would be harder in more bulky devices and can be justified that those patients did not realize bruxism as a serious problem.

Although patient’s recruitment was not very successful, the study gathers a first impressions on the feasibility of an ear device for both circadian cycles in bruxism patients first.

Furthermore, drop-outs could not differentiate whether the pain is caused by bruxism or from another condition, such as muscle-tension because of a disc prolapse in the neck part of the spine. Therefore, such patients could not see the benefit of the device as the pain sensation persisted, despite reporting minor and moderate relief from headaches after using the in-ear device. Five out of seven patients decided to give back their devices within an eight-week testing period because the unpleasant feeling of wearing the device outweighed the unpleasant pain sensation.

Although, there is no proof that the reimbursement policy after giving the device back had negatively affected the fast decision of patients, and the lack of commitment motive should not be neglected.

The study by Tavera et al. [31] showed improvement in all their examined criteria and also there has not been a significant lower efficiency compared to the conventional treatment with night occlusal splint. Therefore, it is to say that Cerezen device does not show inferiority towards splint therapy. Patients reported to be highly satisfied with their devices.

A Google Patents search shows that there has been interest in the field of biofeedback in ear-devices for four decades now. Even though there have been some patents on in-ear devices to treat bruxism, there has only been one product available for purchase so far. As reported by the producer, the product is no longer available with no given reason on why the production was discontinued.

Ideas on devices that create a noise or vibration appear to be promising as new biofeedback intra-aural devices because noise and/or vibration creating devices could make the biofeedback-effect more noticeable for the patient than the biofeedback created by Cerezen™/TMD’es™ inducing pressure on the ear canal wall. The aim of ongoing studies could be to test if noises or vibrations disturb patient’s sleep as well as their everyday-life. However, these ideas give new impulses on how to improve or modify the products that have been available so far to make them more successful and prevent some of their side effects.

There is a need of a new treatment method of bruxism because existing methods like splint-therapy don’t work out for all patients and, as outlined in the instruction, have some negative impact on patients [15]. By testing Cerezen™ in-ear devices we wanted to show if in-ear devices have an improving impact on bruxism patients. We can see in the study, those patients who kept their in-ear devices showed good overall satisfaction and reported improvement on their symptoms like headaches. They value the devices as an invisible and gentle treatment method. However, one of the limitations of this study is the absence of sponsoring that allow the patients to get the devices without any financial loads. The eight-week testing policy of the company where patients can give back the devices and obtain a refund had a negative impact on the results. The expected effects of the devices need more than two months to reach a reasonable treatment outcome. Therefore, a clear and definitive answer of the study question if biofeedback in ear devices are a sufficient method to treat patients with bruxism is challenging. Another reason for the discontinuation of the device can be resembled in the challenge facing new technologies. A dentist might resort to the conventional relieving mechanisms of bruxism, either due to their own belief that conventional methods have proven effective or that patients ask for treatment recommended to bring results by other patients. The understanding of the mechanism of action of the devices and the positive feedback from this study and the other study tested TMD’es™ on 60 patients [31] leads to the conclusion that it is worth to conduct further examinations. A biofeedback mechanism in in-ear devices have a great potential for treating bruxism. The future step in this field is to build on the collected experiences and produce in-ear devices with the help of an acoustician to conduct a prospective clinical full funded study. These may give a perspective collaboration between dentists and acousticians to help patients suffering from bruxism.

**Figure 5:**
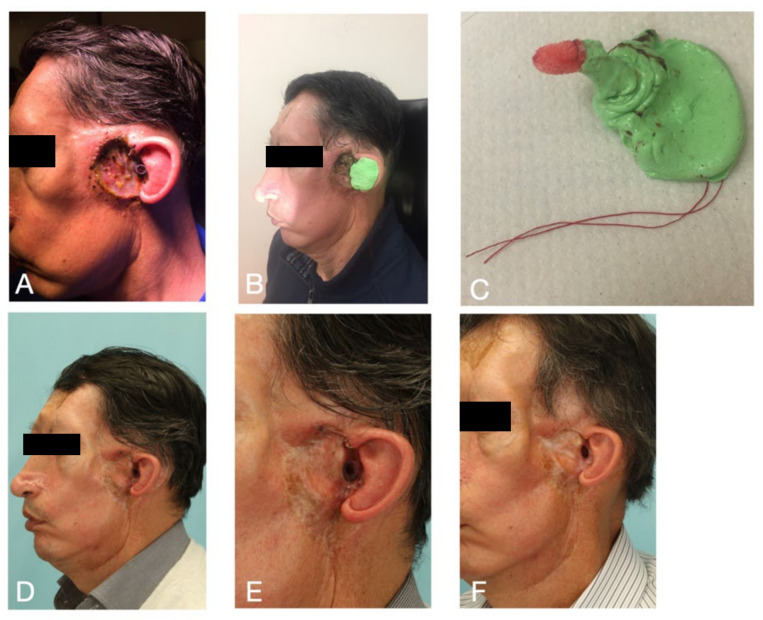
Extended indication for using Cerezen device to obtain an ear canal open till the end of healing process: (**A**) The situation after surgical removal of basal cell carcinoma with part of the external acoustic meatus, the ear canal opened with a plastic foil; (**B**,**C**) taking an impression to produce the in-ear device; (**D**,**E**) the situation one month after using the ear device; (**F**) after complete healing of the ear canal.

In-ear devices can also be implemented to maintain a functional ear canal in patients diagnosed with multiple Basal-cell carcinoma in the face undergo surgical removal of the tumor which infiltrates to the external acoustic meatus. In such cases plastic transparent foil can be unsuccessful method for long time and should be replaced several times. During treatment of a similar case an impression of the operated ear canal was taken to place a Cerezen device which successfully kept ear canal open till the healing was completed (Figure 5). This experience can give the device an extra indication for using in combination with surgical removal in the ear canal region. The most important advantage of this method in comparison with other convectional options is the hollow and rigid design which allow stable opening without hearing impairment.

## 5. Conclusions

Intra-aural devices have great potential to take place in treating bruxism, especially when producers try to reduce the recorded complications in this study and add some functions to build higher awareness of the patient’s habit.

## Figures and Tables

**Table 1:** Details information about the patient base, their age, gender and their initial symptoms. It also shows how long the devices have been worn and whether or not the treatment got discontinued by the patients.

Patient	Age	Gender	Initial Diagnose	Symptomes	Relife of Symptoms	Duration of Usage	Discontinuation of Treatment	First Reported Improvements of the Symptomes
1	58	f	arthrosis	headaches, pain in teeth, pain in periodontal apparatus, pain in muscles, neck pain	yes	2 months	yes	2 months
2	23	f	disc displacement without reduction	headaches, pain in muscles, pain in temporomandibular joint, neck pain	yes	12 months	no	3 months
3	64	f	TMD	muscle pain, pain in temporomandibular joint, pain in teeth and periodontal apparatus, neck pain	yes	11 months	no	3.5 months
4	58	f	TMD	Headaches, pain in teeth, temporomandibular joint, neck pain	No	1 month	yes	No beneficial effect
5	49	f	TMD	headaches, pain in muscles, temporomandibular joint, neck pain	NA	2 months	yes	No effect reported
6	35	f	TMD	headaches, pain in temporomandibular joint, pain in periodontal apparatus, neck pain	yes	6 months	yes	1 month
7	44	f	TMD	pain in temporomandibular joint, neck pain	NA	1 month	yes	No effect reported

## Data Availability

The data presented in this study are available on request from the corresponding author.
